# Surgical repair of aortic dissection 16 years post-Ross procedure

**DOI:** 10.1093/jscr/rjw059

**Published:** 2016-04-30

**Authors:** Mollie R. Myers, J. Trent Magruder, Todd C. Crawford, Joshua C. Grimm, Marc K. Halushka, William A. Baumgartner, Duke E. Cameron

**Affiliations:** 1 Division of Cardiac Surgery, The Johns Hopkins University School of Medicine, Baltimore, MD,USA,; 2 Department of Pathology, Division of Cardiovascular Pathology, The Johns Hopkins University School of Medicine, Baltimore, MD,USA

## Abstract

The Ross procedure is an excellent choice for younger patients in need of aortic valve replacement. While patients have benefited from superior survival rates associated with this procedure, complications related to aortic root dilatation and degeneration of the autograft may be encountered later in life. These challenges may be exacerbated in those with underlying connective tissue abnormalities, a phenomenon commonly observed in the bicuspid aortic valve population. In this report, we present the case of a patient who presented with an aortic dissection 16 years after a Ross procedure for aortic insufficiency in the setting of a bicuspid aortic valve, and review the existing literature related to this adverse event.

## Introduction

The Ross procedure is considered an excellent option for aortic valve replacement in younger patients and in those with a contraindication or aversion to long-term anticoagulation. Ten-year survival for the procedure has been reported as high as 96%, with up to 75% 10-year freedom from reoperation [1]. However, subsequent aortic root dilation and aortic regurgitation are two of the known repercussions of the Ross procedure. One study reported root dilation (>4 cm) in as many as 58% of patients and hemodynamically significant aortic regurgitation in 25% at 7 years [2]. Further research has suggested that connective tissue abnormalities may predispose this population to aneurysmal degeneration and potential aortic dissection, more so than the individual risk attributed to the use of a pulmonary autograft [3]. We present a case in which a patient who had previously undergone a Ross procedure for aortic insufficiency in association with a bicuspid aortic valve presented with a Stanford Type A aortic dissection with involvement of the pulmonary autograft.

## Case

**Figure 1:**
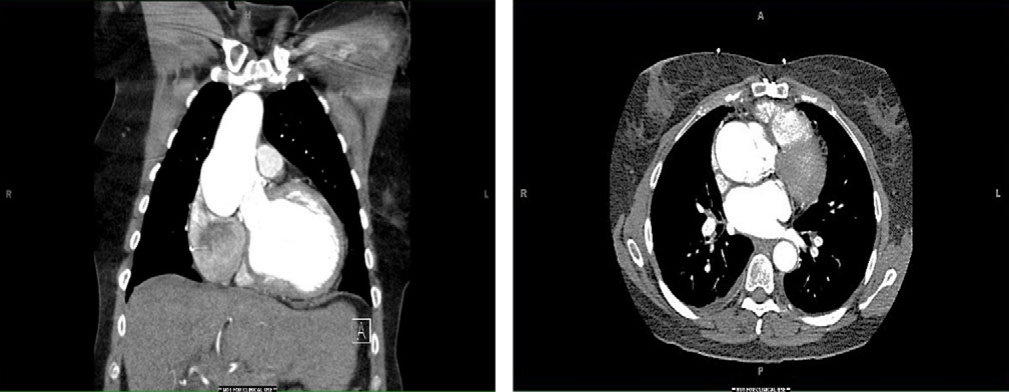
CT of the chest. Coronal (left) and axial (right) views demonstrating pathologic dilatation of the aortic root and ascending aorta. On the axial image, a dissection plane is noted in the aortic root.

A 56-year-old female presented to the emergency department with chest tightness and shortness of breath for 2 days. The patient had a history of a Ross procedure for aortic insufficiency associated with a bicuspid aortic valve 16 years previously. A computed tomography (CT) scan at the time of admission revealed a 6.1-cm aneurysm with a small dissection flap (Fig. 1). The patient had recently undergone an echocardiogram that revealed a preserved ejection fraction of 55–60% and aortic root dilation to 5.9 cm with additional dilation of the ascending aorta to 4.4 cm. As echocardiography just 6 months earlier had measured the aortic root and ascending aorta at 5  and 3.8 cm, respectively, concern had been raised for accelerated aneurysmal degeneration and planning for elective aneurysm repair had been underway. Given the patient’s symptomatic presentation and associated CT findings, she was admitted for medical optimization and was taken to the operating room 8 days later.

**Figure 2:**
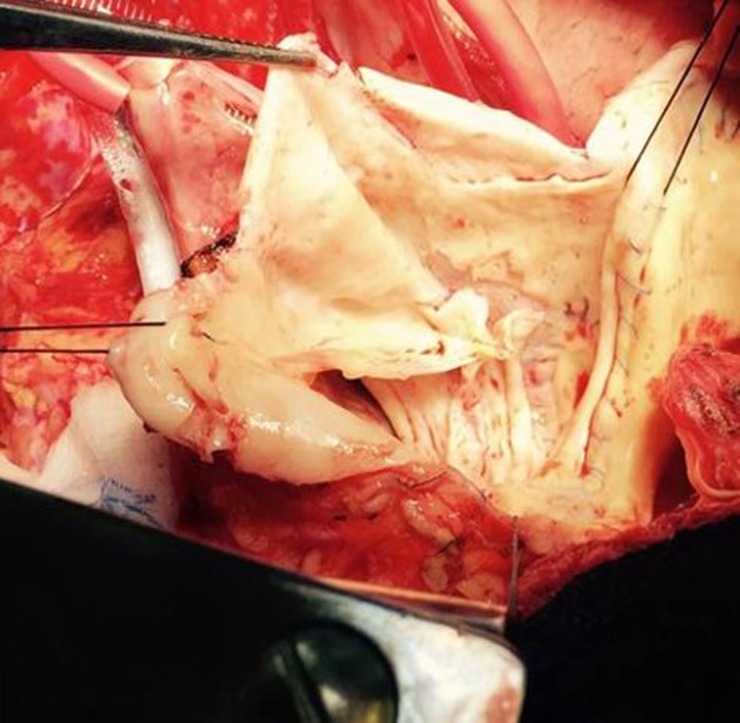
Gross image of the pulmonary autograft in the aortic valve position with significant degeneration of the valve leaflets and a dissection plane (top portion of picture) extending into the non-coronary sinus.

Intraoperatively, transesophageal echocardiography revealed severe aortic regurgitation with dilatation of the autograft root to 6.4 cm. On gross examination, degeneration of the aortic valve leaflets and limited dissection of the non-coronary sinus of the autograft was appreciated (Fig. 2). After the autograft and valve were excised and the coronary artery buttons mobilized, a 29-mm bioprosthesis and Valsalva graft were sutured to the annulus of the autograft. The coronary buttons were anastomosed to the graft, and finally the graft was anastomosed to the distal ascending aorta. After routine de-airing maneuvers, the aortic cross-clamp was released, and immediately the patient experienced severe left ventricular (LV) dysfunction and hemodynamic instability. Accordingly, the heart was rearrested and the Valsalva graft was opened to inspect the aortic valve and coronary buttons. This revealed significant narrowing of the ostium of the left coronary button, and this anastomosis was subsequently revised. An intra-aortic balloon pump was placed via the left femoral artery. The patient was warmed and weaned from bypass; however, the sternum was not closed out of concern for ongoing LV dysfunction. After 2 days in the ICU, the patient stabilized with improvement in LV function and returned to the operating room for IABP removal and sternal closure. After an uneventful remaining course, the patient was discharged to home 9 days later.

## Discussion

Limited data exist with regard to aortic dissections that occur subsequent to a Ross procedure. However, among patients who have undergone conventional aortic valve replacement, the incidence of dissection approaches 27% in patients with aortic dilation >5.0 cm, as compared to an incidence of 0.6% overall [4]. To the best of our knowledge, only four cases of aortic dissection following the Ross procedure have been reported. One involved a patient with a Debakey Type II dissection 6 years postoperatively, which was repaired with a Dacron tube graft [5]. Another patient suffered an aortic dissection 7 years after a Ross procedure, which was repaired utilizing the Yacoub technique (remodeling), again with a Dacron graft [6]. The other two cases involved a 50-year-old woman and a 50-year-old man who suffered dissections at 9 and 5 years post-Ross procedure, respectively [7, 8]. Our case is unique in that the event of dissection was temporally separated from the initial Ross procedure by a longer time interval, 16 years.

**Figure 3:**
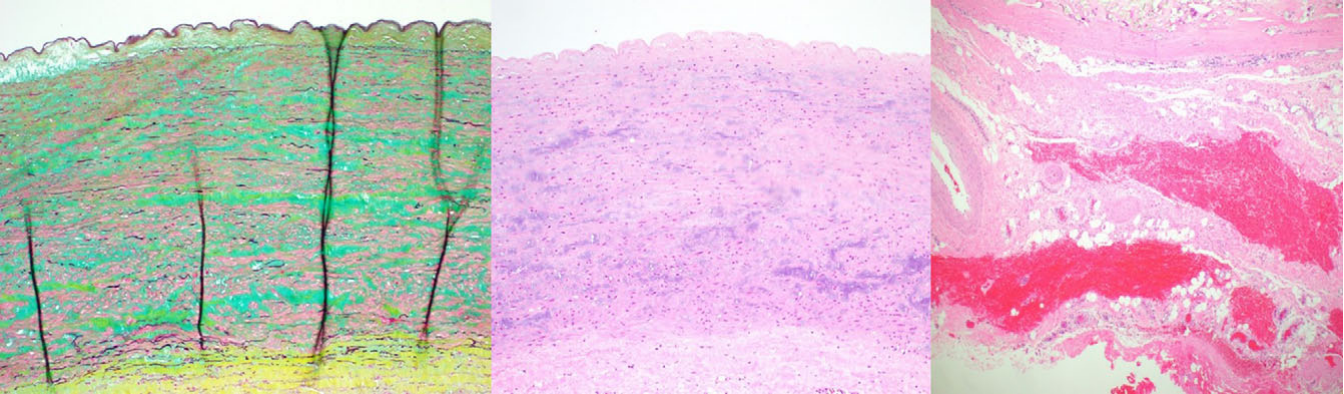
Histologic images from the dissected pulmonary autograft. Left: degradation of the media with translamellar mucoid extracellular matrix accumulation and fragmentation and/or loss of elastic fibers; middle: loss of smooth muscle nuclei with mild smooth muscle cell disorganization; right: Adventitial hemorrhage consistent with an aortic dissection is present in the lower portion of the image (Movat Pentachrome magnification, *x*10, H&E *x*10 and H&E *x*4).

Pertinent to our patient, whose original aortic valvulopathy was related to a native bicuspid aortic valve, is the question of whether the aneurysmal changes of the autograft were also mediated by connective tissue abnormalities. In fact, pathological examination of this patient’s operative specimen revealed medial degeneration with mucoid extracellular matrix accumulation, fragmentation of elastic fibers and loss of smooth muscle nuclei (Fig. 3), consistent with an underlying connective tissue disorder. Indeed, studies have identified histopathological irregularities in patients with a bicuspid aortic valve. One study of bicuspid aortic valve patients found that almost half had degenerative changes in the aorta (cystic medial necrosis, fragmentation and loss of elastic fibers and deposition of mucopolysaccharide material), and 75% had pulmonary trunk degeneration [3].

Other studies have shown that as many as 13% of patients with an aortic dissection have a concomitant congenital defect of the aortic valve [9]. Further evidence of a genetic contribution is suggested by reports of familial bicuspid aortic valve disease. In these families, an increased incidence of dilation and dissection has been observed [10]. A defect in neural crest maturation has been proposed as a potential mechanism of aortopathy, recognizing that both the arterial media and aortic valve cusps are derived from neural crest origin [10].

In conclusion, the Ross procedure remains a reasonable choice for aortic valve replacement in younger patients. However, enthusiasm for this autograft must be tempered in the bicuspid aortic valve population, given the association with connective tissue aberrations that may predispose this patient group to subsequent aortopathy.

## Conflict of Interest Statement

None declared.
